# Research Progress on the Potential Pathogenesis of Persistent Postural–Perceptual Dizziness

**DOI:** 10.1002/brb3.70229

**Published:** 2024-12-31

**Authors:** Chen Qin, Ruyi Zhang, Zhihui Yan

**Affiliations:** ^1^ Department of General Practice Yantaishan Hospital Affiliated to Binzhou Medical University Yantai China; ^2^ Department of Cardiology Yantaishan Hospital Affiliated to Binzhou Medical University Yantai China

**Keywords:** anxiety, cerebral blood flow, functional connectivity, persistent postural–perceptual dizziness

## Abstract

**Introduction:**

Persistent postural–perceptual dizziness (PPPD) is the most prevalent chronic functional dizziness in the clinic. Unsteadiness, dizziness, or non‐spinning vertigo are the main symptoms of PPPD, and they are typically aggravated by upright posture, active or passive movement, and visual stimulation. The pathogenesis of PPPD remains incompletely understood, and it cannot be attributed to any specific anatomical defect within the vestibular system. Consequently, there is no objective examination method for the disease, and the diagnosis primarily depends on the symptoms of the patient, which lack specificity.

**Methods:**

To better understand the pathogenesis of PPPD and to aid in the development of novel diagnostic strategies and therapies, we conducted a comprehensive narrative review of the relevant literature. We performed a search for literature in PubMed using the following search phrases: “persistent postural–perceptual dizziness” OR “PPPD” OR “chronic subjective dizziness” OR “functional dizziness” OR “space‐motion discomfort” OR “visual vertigo” OR “phobic postural vertigo.” The reference list of relevant studies was also screened. The search was limited to publications in English, and the final references were selected based on their relevance to the scope of this review.

**Results:**

This review summarizes recent studies that have investigated the pathogenesis of PPPD. It is traditionally assumed that PPPD may result from altered postural control strategies, cortical integration of threat assessment and spatial orientation, or abnormal integration of multi‐sensory information. Recent studies have shown that the brain structure, activity, structural connectivity, and even cerebral perfusion of patients with PPPD differ from those of healthy individuals. Furthermore, PPPD patients are different from healthy individuals in spatial navigation ability, vestibular perception thresholds, central sensitization, and oxidative stress. These findings provide additional anatomical and behavioral insights into the pathogenesis of PPPD, suggesting that PPPD may arise from shifts in the interactions among emotional, visuo‐vestibular, and sensorimotor networks.

**Conclusion:**

Understanding the complex pathogenesis of PPPD is crucial for the development of novel therapeutics against PPPD. Following the existing findings, our review suggests directions for future research.

## Abstract 2

This review has summarized recently published studies of PPPD to provide a comprehensive analysis of the specific pathogenesis underlying PPPD. A full understanding of the pathogenesis of PPPD will help to find novel therapeutic targets.

## Graphical abstract

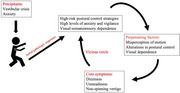

**Abbreviations**
ACC: anterior cingulate cortex
ALFF: amplitude of low‐frequency fluctuations
CSD: chronic subjective dizziness
DHI: Dizziness Handicap Inventory
fALFF: fractional amplitude of low‐frequency fluctuation
FC: functional connectivity
fMRI: functional magnetic resonance imaging
GMV: gray matter volume
IFg: inferior frontal gyrus
LGI: local gyrification index
MRI: magnetic resonance imaging
PIVC: parieto‐insular vestibular cortex
PPPD: persistent postural–perceptual dizziness
rCBF: regional cerebral blood flow
SPECT: single photon emission computed tomography
STG: superior temporal gyrus
TPJ: temporal–parietal junction

## 1 Introduction

The diagnostic criteria for persistent postural–perceptual dizziness (PPPD) were released in 2017 by the Bárány Society (Staab et al. 2017), which were based on a comprehensive review of three decades of study on phobic postural vertigo, space–motion discomfort, visual vertigo, and chronic subjective dizziness (CSD). PPPD is a prevalent chronic functional dizziness in clinics. The core symptoms of PPPD are unsteadiness, dizziness, and non‐spinning vertigo. The symptoms present on most days for 3 months or longer and can be aggravated by upright posture, active/passive movement, and visual stimulation. Minor illnesses can impact the patient's quality of life, whereas severe illnesses may prevent them from working, imposing a significant burden on both family and social healthcare resources (Azzi et al. 2022; Mueller et al. 2014; Ruthberg et al. 2021). Compared to other patients with dizziness, those with PPPD experience a significantly greater burden of dizziness and a reduced physical quality of life (Steensnaes et al. 2023; Teh and Prepageran 2022).

**FIGURE 1:**
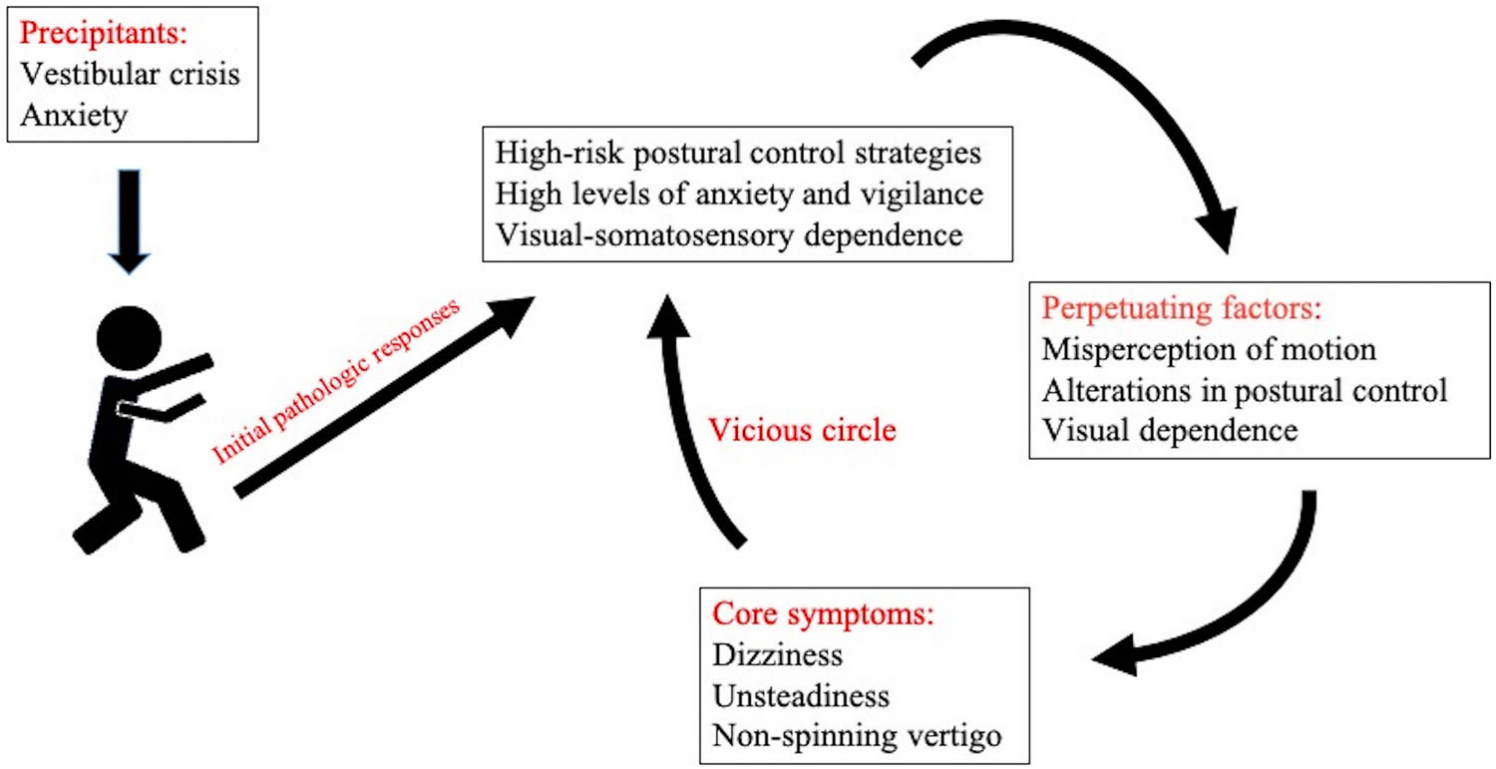
A schematic overview of the occurrence and development of PPPD.

There are relatively scarce randomized controlled trials on PPPD, and its pathogenesis remains poorly understood. This limits the development of diagnostic and therapeutic techniques for PPPD. Most of the understanding of its pathogenesis is based on its four predecessors. Current hypotheses suggest that the pathogenesis of PPPD involves multiple factors, such as anxiety‐related personality traits, altered postural control strategies, visual dependence, and abnormal integration of multi‐sensory information (Staab et al. 2017). The present treatment options encompass patient education (Seemungal and Passamonti 2018), vestibular rehabilitation therapy (Ibrahim et al. 2023; Mempouo et al. 2021; Nada, Ibraheem, and Hassaan 2019; Teh et al. 2023), cognitive behavior therapy (Herdman et al. 2022; Waterston et al. 2021; Yu et al. 2018; Zhao et al. 2022), medication (Min, Kim, and Park 2021; Miwa and Kanemaru 2022; Staab, Ruckenstein, and Amsterdam 2004), electrical stimulation (Eren et al. 2018; Im et al. 2022), and combination therapy (Axer et al. 2020), which lack specificity. Thus, we provide an overview of recently published studies of PPPD to present a comprehensive analysis of the specific pathogenesis underlying PPPD. Figure 1 displays a schematic overview of the occurrence and development of PPPD. A full understanding of the pathogenesis of PPPD will help to find novel therapeutic targets.

## 2 Potential Pathogenesis

### 2.1 Anxiety‐Related Factors

#### 2.1.1 Anxiety‐Related Personality Traits May Be an Important Risk Factor for PPPD

PPPD can be triggered by disorders that cause dizziness/vertigo and balance disorders (Trinidade et al. 2023). Common triggering events include acute central or peripheral vestibular disorders (25%), vestibular migraines (15%–20%), and panic attacks (15%–20%) (Staab 2020). People with anxiety‐related personality traits are at a higher risk of suffering from CSD (Staab et al. 2014) (a predecessor of PPPD) and PPPD (Yan et al. 2017). Anxiety‐related medical history and family history are similarly predisposing factors for CSD (Staab and Ruckenstein 2003) or persistent dizziness (Best et al. 2009) after precipitating events. In contrast, patients with a personality of optimism and high resilience are less likely to suffer from persistent dizziness following an acute vestibular episode (Tschan et al. 2011).

PPPD and anxiety disorders share many behavioral symptoms, such as avoidance, social withdrawal, hyperarousal, or neurological symptoms such as dizziness and postural and gait disturbances (Maywald et al. 2023). Several recent studies have supported the importance of anxiety in the development of PPPD. Trinidade et al. (2020) found that PPPD patients showed lower conscientiousness, higher introversion, higher neuroticism, and higher anxiety compared to non‐dizzy patients. Kitazawa et al. reported that the Niigata PPPD Questionnaire score of PPPD patients was notably higher than that of those with undifferentiated dizziness and unilateral vestibular hypofunction. Moreover, the anxiety score on the hospital anxiety and depression scale of PPPD patients was significantly higher than that of those with undifferentiated dizziness (Kitazawa et al. 2021). According to a Chinese study, the prevalence of anxiety problems among patients with PPPD was found to be as high as 48.57% (Yang, Ding, and Wu 2021). Similarly, another Chinese study including 122 PPPD patients showed that 46.72% of the patients had emotional disorders, and the ratio of anxiety to depression was 3.1:1 (Zhang et al. 2022). Patients with primary‐PPPD had a higher probability of anxiety than those with secondary‐PPPD. In summary, these results indicate that anxiety is more common among patients with PPPD, which is consistent with conventional wisdom. In the evolution of PPPD, a highly anxious response to a trigger event is possibly the most critical initial pathophysiologic response to the disease.

However, the studies conducted by Trinidade et al. and Kitazawa et al. were retrospective and included PPPD patients with different courses of the disease. This heterogeneity may have affected the research conclusions. The latter two Chinese studies, although prospective, exclusively enrolled hospitalized patients, so it is possible that patients with more serious conditions were selected for. This selection bias has reduced the reliability of their research conclusions. Furthermore, the view of another study is contrary to the findings above. The study by Li et al. (2020b), involving 10 PPPD patients, found no significant anxiety or depression in any of the patients. Depression and anxiety may be just complications of PPPD, not its primary risk factors. However, the sample volume of the present study is not large enough, and a larger sample size is required to validate this view.

#### 2.1.2 Anxiety‐Related Hormones and Neurotransmitters Alter in PPPD

Dopamine receptor genes are the closest receptor genes to personality traits. Polymorphism in the Dopamine D2 receptor gene is associated with neurotic personality (Kazantseva et al. 2011), which is closely related to anxiety. Through a negative feedback regulation mechanism, the dopamine receptor controls the concentration of dopamine in synapses (Brake et al. 2004). Cui et al. (2019) demonstrated that the PPPD group had a higher allele frequency for the A1 allele than the control group. As a result, there could be fewer dopamine receptor D2 molecules in the relevant brain regions of PPPD patients, especially in the striatum. Additionally, there could be a greater tendency to develop a neurotic personality in the occurrence of acute vestibular vertigo or other environmental stimulus conditions in PPPD patients compared with controls. The A2/A2 genotype contributes to preventing the development of PPPD, whereas the A1 allele may be a susceptibility gene for PPPD. DNA methylation is one of the epigenetic modification methods, and it may be another pathogenesis of PPPD. However, the sample size of the study was small, and the subjects were all Chinese. There are significant genetic differences among ethnic groups. Therefore, more studies with larger samples or in other countries are needed to confirm this conclusion.

Cortisol, adrenaline, and serotonin are also emotional stress‐related hormones. Fang et al. (2020) studied serum samples from patients with CSD. Compared to healthy controls, CSD patients had significantly higher serum cortisol and adrenaline levels. The serum serotonin level of patients with CSD was significantly lower than that of healthy people. The dysregulation of hormone or neurotransmitter levels is involved in the pathophysiological process of anxiety in CSD patients. However, the study was conducted in patients with CSD rather than PPPD, so it is unclear whether this conclusion applies to PPPD patients. Further studies are needed to investigate the relationship between the levels of anxiety‐related hormones and neurotransmitters in PPPD patients and the occurrence and development of the disease. Such changes in hormones and neurotransmitters associated with anxiety in PPPD may help us identify patients who are more likely to develop PPPD in the early stage of acute vestibular disorder. The incidence of PPPD may be decreased by early detection of high‐risk individuals and the use of suitable therapies, such as cognitive behavior therapy. We hope that there will be relevant clinical studies to confirm this hypothesis.

#### 2.1.3 Changes in the Function of Anxiety‐Related Neural Networks in PPPD

There is an overlap between the central vestibular pathway and anxiety‐related neural networks in healthy individuals. The neural plasticity of the limbic and prefrontal cortices is impacted by anxiety (Christoffel, Golden, and Russo 2011; McEwen et al. 2012). The neural cortex associated with anxiety includes the insula, anterior cingulate gyrus, medial prefrontal cortex, and amygdala (Paulus 2008). Previous studies have reported that personality traits such as neuroticism may have an impact on major visuo‐vestibular and anxiety networks in the brain, which may be the root of PPPD (Indovina et al. 2014; Riccelli et al. 2017). A study on neuroticism and brain function in PPPD patients showed that neuroticism improved connectivity between the occipital regions and inferior frontal gyrus (IFg) and increased activity in the IFg during vertical motion (Passamonti et al. 2018). These results suggest that anxiety‐related neural networks are crucial in PPPD and provide a theoretical basis for the application of transcranial direct current stimulation (Im et al. 2022) or transcranial magnetic stimulation therapy (Staab 2023) in the prefrontal cortex.

On the basis of the pathogenesis above, the most commonly used pharmacotherapy for PPPD at present is anti‐anxiety therapy. Anti‐anxiety medications include selective serotonin reuptake inhibitors and selective serotonin norepinephrine reuptake inhibitors. Randomized controlled trials are needed to select the most effective drugs with minimal side effects.

### 2.2 High Levels of Anxiety and Vigilance About Acute Symptoms During Precipitating Events

High levels of anxiety and vigilance about dizziness during and after episodes of acute vestibular disease are predictive of persistent dizziness months later in several prospective studies (Cousins et al. 2017; Godemann et al. 2005; Heinrichs et al. 2007). In addition, such kinds of emotional responses may be the initial pathologic responses in the development of PPPD. The specific mechanisms through which balance vigilance affects PPPD remain unknown. Some researchers have hypothesized that patients with high balance vigilance become consciously sensitized to minor discrepancies between predicted and actual postural movements. They make a more conscious effort to maintain control of posture, which disrupts normal readaptation and initiates a vicious cycle of maladaptation. This ultimately leads to the occurrence of PPPD. A case–control pilot study showed that PPPD patients had increased body vigilance to dizziness than both recovered vestibular patients and non‐dizzy patients, and the illness perceptions of PPPD patients indicated higher levels of threat than recovered vestibular patients (Trinidade et al. 2020). The evidence above reveals that these early psychological reactions have significantly stronger impacts on long‐term outcomes than the initial acute physical symptoms of patients. However, the sample size of this exploratory study was particularly small, and it was retrospective, making the conclusions unconvincing. Extensive research of this phenomenon in the early stages following vestibular injury is required to validate this point.

The Balance Vigilance Questionnaire has been developed for assessing vigilance toward balance, which has been confirmed to be a valid and reliable self‐report instrument (Ellmers and Kal 2024). Clinicians can use this questionnaire to assess the bodily vigilance of patients after an acute vestibular disorder. Screening patients with high balance vigilance as early as possible and giving a targeted treatment can help to reduce the possibility of such patients developing PPPD. A retrospective review of 198 PPPD patients evaluated the effects of cognitive behavior therapy intervention (Waterston et al. 2021). The results of the study indicate that cognitive behavior therapy can reduce dizziness symptoms, anxiety, avoidance, and safety behaviors, as well as disability in PPPD patients over up to 6‐month follow‐up periods. The effectiveness of cognitive behavior therapy reveals the important role of high levels of anxiety and vigilance in the development of PPPD. The weakness of this study was the lack of a control group. Therefore, whether the improvements were due to cognitive behavior therapy or placebo effects is unclear. This simple and cost‐effective therapeutic measure deserves further exploration in PPPD.

### 2.3 Alterations in Postural Control Strategies

Healthy people often use high‐risk postural control strategies in response to vertigo or dizziness, such as stiffening up and taking shorter steps in heights and slippery environments (Adkin et al. 2000). After the threat has attenuated, these postural control strategies will stop. However, in PPPD patients, acute vestibular events induce the use of high‐risk postural control strategies. In addition, initial high levels of anxiety and vigilance about acute symptoms seem to perpetuate the process in an attempt to adjust to the perceived postural threat that has vanished. This could be attributed to a lower threshold for the activation of closed‐loop feedback control mechanisms, which are commonly used for demanding locomotor tasks (Wuehr et al. 2013). Such a pathophysiologic process leads to maladaptation in PPPD patients (Staab 2020) and is considered to be one of the most important pathogenesis of PPPD (Staab et al. 2017).

Physiologic studies of patients with PPPD and its predecessors have identified that alterations in postural control include low‐amplitude and high‐frequency postural sway (Odman and Maire 2008; Wuehr et al. 2013). The performance of PPPD patients and healthy controls was compared during challenging balance tasks to look into postural control in PPPD (Lubetzky et al. 2021). PPPD group had a larger center of pressure path in the static visual scene condition in the antero‐posterior direction. In addition, in the static visual scene as well as the other two more challenging balance tasks, the center of pressure acceleration of the PPPD group was considerably higher. High center of pressure acceleration in the anterior–posterior direction was thought to reflect the increased effort of the muscles surrounding the ankle to maintain a stable position by preventing excessive movement of the upper segments. These results agree with a postural control strategy in PPPD, that is, co‐contraction of lower limb musculature. Moreover, a cross‐sectional prospective study assessed the static balance and postural sway in both healthy people and PPPD patients while standing with eyes open or closed on a firm or foam surface (Anagnostou et al. 2021). Patients with PPPD showed distinct variations in spectral content under equal sway area sensory feedback conditions. There was decreased high‐frequency and increased low‐frequency sway in PPPD patients. Meanwhile, the Dizziness Handicap Inventory (DHI) score showed a positive correlation with middle‐frequency fluctuations and a negative correlation with low‐frequency. The above two studies attempted to quantify the postural control strategy in PPPD patients using time and frequency domain analysis. They all found that PPPD patients had more rigid postural control strategies than healthy controls. However, the two studies failed to verify each other due to differences in research methods, intervention measures, and observation indicators. In addition, they only compared PPPD patients with healthy controls, not with other vestibular disorders, so it is difficult to determine whether these changes in postural control strategy are unique to PPPD. In the future, it is essential to combine the time domain and frequency domain balance parameters to evaluate the posture of PPPD patients, which can interpret the postural control strategy of PPPD patients in more dimensions. Furthermore, it will be meaningful to compare the postural control strategy of PPPD with other vestibular disorders, which will help to find the discrimination point between PPPD and other vestibular disorders from the perspective of posture.

The study conducted by Kobel, Wagner, and Merfeld (2023) is of pioneering significance as it is the first to evaluate nonlinear measures in adults who meet the diagnostic criteria for PPPD. They assessed recurrent quantification analysis to figure out if the dynamic structure of sway changed in adults with PPPD. According to their findings, patients with PPPD generally showed more repeatable and simple postural control behaviors. This information indicates that the posture control system of PPPD patients is more rigid and less adaptable. This inspires us that nonlinear measures can offer distinctive insights into how PPPD affects postural control. Therefore, quantification analysis may be an effective diagnostic method or rehabilitation outcome measurement tool for PPPD. However, this study had limitations. A wide age range might interfere with the results due to physiological differences among different age groups. Moreover, a high proportion of patients with vestibular migraine might reduce the representativeness of pure PPPD patients. Therefore, the conclusions may be biased. In addition, another research study found a disproportionate increase in the perceived sway compared to the actual sway in patients with PPPD (San Pedro Murillo et al. 2023). This may result from the mismatch between “bottom‐up” inputs and maladaptive signals from “top‐down” attentional control systems (Seemungal and Passamonti 2018). Metrics of both perceptual impairment and postural sway outcomes may be biomarkers of PPPD. Future studies are necessary to explore the relationship between these metrics and the pathogenesis of PPPD to optimize diagnosis and rehabilitation strategies.

### 2.4 Visual Dependence

Normal postural stability occurs due to the accurate processing of signals from the motor organs by the central nervous system and the balance among the sensory inputs of visual, vestibular, and somatosensory information. The tendency to rely more heavily on visual information than on vestibular or somatosensory information to determine spatial orientation is known as visual dependence. This results in individuals being unable to respond appropriately to self‐motor feedback. The pathophysiology of visual dependence remains unclear. Several previous studies on predecessors of PPPD have shown that visual dependence is one of their most important characteristics (Bronstein 1995; Guerraz et al. 2001; Pavlou, Davies, and Bronstein 2006; Redfern, Furman, and Jacob 2007).

Studies have investigated the visual dependence of patients with PPPD in the past few years. One hundred and eighteen untreated PPPD patients completed the Niigata PPPD Questionnaire, and factor analysis was performed on answers to the questionnaire (Yagi et al. 2021). It is demonstrated that the sensitivity to visual stimulation was the primary exacerbating factor of PPPD. In addition, visual dependence tests were conducted among healthy individuals, dizzy non‐PPPD patients, and PPPD patients (De Vestel et al. 2022). PPPD patients showed greater visual dependence, without a decline in postural balance. The study also revealed that the most prominent feature of PPPD in patients with chronic dizziness was elevated Visual Vertigo Analog Scale scores. The same results were also found in a recent study using foam photography (Ichijo et al. 2024). Patients with PPPD had significantly higher Romberg's ratios than controls. In addition, PPPD patients with normal vestibular function also had higher Romberg's ratios than controls. This suggests that visual dependence is an important feature of PPPD and not affected by vestibular function. All the above clinical studies have verified that PPPD patients are more dependent on visual input, and visual dependence is one of the crucial potential pathogenesis of PPPD. Although the results of Yagi et al.’s study were based on a questionnaire survey, which was somewhat subjective, existing neuroimaging studies have confirmed the above views from an objective perspective.

Visual cortex activity was measured using functional magnetic resonance imaging (fMRI) during a virtual‐reality rollercoaster simulation in PPPD patients (Riccelli et al. 2017). Dizziness handicap was positively correlated with increased activity in the visual cortex among patients diagnosed with PPPD. The degree of visual dependence may be substantially related to the intensity of symptoms in patients with PPPD. This provides a new target for the treatment of PPPD. We can provide a tailored vestibular rehabilitation program for patients with visual dependence. For example, we can have patients observe complex visual backgrounds such as jagged striped pictures and rotating striped umbrellas. Although this process may have mild symptoms, it can promote balance compensation through habituation. In addition, the virtual‐reality system has a good function in promoting vestibular adaptation, reducing visual sensitivity, reducing symptoms of dizziness, and improving the quality of life (Choi et al. 2021; Mempouo et al. 2021). Exposing patients to symptom‐inducing visual motion in such a gradual manner can reduce patients’ reliance on visual information and increase the weight of vestibular–somatosensory information.

Visual dependence can lead to the exacerbation of PPPD when exposed to complex visual stimulation, which is called visual exacerbation. Once visual exacerbation occurs, symptoms of PPPD may persist for an extended period (Staab et al. 2017). Studies have indicated that PPPD patients experience visual exacerbation due to neural mechanisms associated with gaze instability (Schröder et al. 2021; Yagi et al. 2022). Although gaze stabilization driven by sensory input remained intact, there were significant and quantifiable deficits in internally driven gaze stabilization in individuals with PPPD. This indicates a discrepancy between internal expectations and real bodily states in PPPD (Schröder et al. 2021). Moreover, individuals with PPPD were more likely to exhibit gaze instability after being exposed to dynamic visual stimulation than individuals with unilateral vestibular hypofunction and healthy controls, which may exacerbate vestibular symptoms (Yagi et al. 2022). This finding could illuminate the neural mechanisms of visual exacerbation in PPPD patients. However, how this pathophysiological mechanism affects symptom perception remains to be seen. Gaze instability provides a measurable positive diagnostic modality for PPPD.

### 2.5 Changes in Brain Structure and Function

Recent progress in neuroimaging has brought new information about the structure and functional connectivity (FC) of the brain in PPPD patients, as vestibular evaluation, clinical laboratory testing, and physical examination are always normal in PPPD. Significant differences have been observed in the brain structure, brain activity, brain FC, and regional cerebral blood flow (rCBF) between PPPD patients and healthy controls. Table 1 shows neuroimaging studies in patients diagnosed with PPPD available at present.

**TABLE 1:** Neuroimaging studies in patients diagnosed with persistent postural–perceptual dizziness (PPPD).

Study	*N* (patients:controls)	Modality	Dizziness scale	Results in PPPD patients	Association between imaging changes and dizziness
Wurthmann et al. (2017)	42:42	MRI	DHI	Gray matter volume decreased in the dorsolateral prefrontal cortex, precentral gyrus, temporal cortex, cingulate cortex, hippocampus, caudate nucleus, and cerebellum	Gray matter volume in the somatosensory processing structures, supplementary motor area, and visual cortex was negatively correlated with the disease duration
Riccelli et al. (2017)	15:15	Task‐based fMRI	DHI	When moving vertically as opposed to horizontally, the central insular sulcus did not become more active	There was a positive correlation between dizziness handicap and increased activity in the visual cortex bilaterally
Nigro et al. (2019)	15:15	MRI	DHI	Significant reductions in LGI were observed in bilateral multi‐modal vestibular regions	There was a positive correlation between dizziness severity and LGI in visual areas, as well as a negative correlation in the right superior parietal cortex between dizziness severity and LGI
Lee et al. (2018)	38:38	Resting‐state fMRI	DHI	There were increased FC of the subcallosal cortex with left middle frontal gyrus and left superior lateral occipital cortex. There were decreased FC of the left hippocampus with right insular cortex, left posterior opercular cortex, bilateral central opercular cortices, and cerebellum, and decreased FC between anterior left temporal fusiform cortex and right nucleus accumbens	PPPD patients showed a positive correlation between dizziness handicap and FC among the bilateral supramarginal gyrus and left posterior lobes of the cerebellum, as well as connectivity centered on the cerebellar lobule X and the right inferior temporal gyrus. Furthermore, negative correlations were observed between dizziness handicap and the FC of the left amygdala, left parietal opercular cortex, and left hippocampus

Study	*N* (patients:controls)	Modality	Dizziness scale	Results in PPPD patients	Association between imaging changes and dizziness
Na et al. (2019)	25:25	SPECT	DHI	PPPD patients showed decreased rCBF in the frontal lobe and insula. They also showed increased rCBF in the cerebellum bilaterally	No correlation
Passamonti et al. (2018)	15:15	Task‐based fMRI	DHI	There was a positive correlation between neuroticism and activity in the IFg. In addition, there was enhanced FC between the IFg and associative visual regions in response to vertical versus horizontal motion	Not mentioned
Li et al. (2020a)	12:12	Resting‐state fMRI	DHI	PPPD patients showed decreased intra‐network FC in the right precuneus, between right precuneus and bilateral precuneus and left premotor cortex. They also showed increased inter‐network FC between the occipital pole visual network and lateral visual and auditory networks, as well as the auditory, sensorimotor networks	PPPD patients showed a negative correlation between FC changes and DHI functional scores
Li et al. (2020b)	10:10	Resting‐state fMRI	DHI	PPPD patients showed lower regional homogeneity values and ALFF in the cuneus and right precuneus. Further seed‐based FC analysis showed decreased FC between the left precentral gyrus, cuneus and precuneus	Not mentioned

Study	*N* (patients:controls)	Modality	Dizziness scale	Results in PPPD patients	Association between imaging changes and dizziness
von Söhsten Lins et al. (2021)	16:16	Task‐based fMRI	DHI	In response to negative versus positive stimuli, PPPD patients showed decreased activity in ACC and increased activity in left angular gyrus. In negative versus neutral and negative versus positive contrasts, PPPD patients showed increased activity in intraparietal sulcus and parahippocampal gyrus	Not mentioned
Jiang et al. (2022)	18:18	Multifrequency Magnetoencephalography	DHI	PPPD patients showed a significant increase in neuromagnetic activity in the TPJ at 1–4 Hz and 4–8 Hz and a significant increase in the frontal cortex at 1–4 Hz	PPPD patients showed a positive correlation between localized source strength in TPJ at 1–4 Hz and the DHI score
Yagi et al. (2023)	11:11	Task‐based fMRI, resting‐state fMRI	DHI	Compared with the pre‐stimulus condition, PPPD patients showed increased FC between the spatial cognitive areas and visual areas and that between the prefrontal and visual areas in the post‐stimulus condition	Not mentioned
Liu et al. (2024)	30:30	Resting‐state fMRI	DHI	PPPD patients showed increased fALFF in the right precuneus and decreased voxel‐mirrored homotopic connectivity in the bilateral precuneus	PPPD patients showed a positive correlation between precuneus fALFF values and DHI scores, and a negative correlation between voxel‐mirrored homotopic connectivity values and the disease duration

Abbreviations: ACC, anterior cingulate cortex; ALFF, amplitude of low‐frequency fluctuations; DHI, Dizziness Handicap Inventory; fALFF, fractional amplitude of low‐frequency fluctuation; FC, functional connectivity; fMRI, functional magnetic resonance imaging; IFg, inferior frontal gyrus; LGI, local gyrification index; MRI, magnetic resonance imaging; rCBF, regional cerebral blood flow; SPECT, single photon emission computed tomography; TPJ, temporal–parietal junction.

#### 2.5.1 Changes in Structure

Up to now, two structural MRI studies have been performed to figure out the structural alterations in the brain associated with PPPD (Nigro et al. 2019; Wurthmann et al. 2017). Wurthmann et al. used voxel‐based morphometry on T1‐weighted images to examine differences in regional gray matter volume (GMV) between PPPD patients and healthy controls (Wurthmann et al. 2017). In this study, reduction of GMV was identified in the left anterior cingulate cortex (ACC), the left‐sided posterior hippocampus, the left superior temporal gyrus (STG), the left motion‐sensitive area, the right precentral gyrus, bilateral middle temporal gyrus, bilateral cerebellum, as well as the dorsolateral prefrontal cortex and the left sides of the caudate nucleus in PPPD patients compared with controls. Moreover, no increase in GMV was observed in PPPD patients. Notably, regression analysis revealed an inverse correlation between disease duration and GMV in secondary and associative visual cortices, as well as in the supplementary motor area and bilateral postcentral gyrus. The findings of this study are in contrast to the previous research that validated elevated cerebral GMV in patients with phobic postural vertigo (Popp et al. 2018). This may provide further evidence that PPPD and phobic postural vertigo are fundamentally distinct diseases.

In another structural MRI study, surface‐based morphometry was employed to examine structural changes in PPPD (Nigro et al. 2019). Patients with PPPD showed a significant reduction in local gyrification index (LGI) in bilateral multimodal vestibular regions, particularly in the posterior insula, supramarginal gyrus, and posterior STG. LGI is the most widely used indicator of cortical folding and is capable of quantifying the amount of cortical surface invaginated in the cerebral sulci (Schaer et al. 2008). Cortical folding indicates the mechanical stress along the axons connecting different locations (Van Essen 1997). Therefore, decreased cortical folding indicates reduced connections within the white matter. In addition, in the PPPD group, there was a positive correlation between dizziness severity and LGI in visual areas, as well as a negative correlation between dizziness severity and LGI in the right superior parietal cortex.

Overall, the two structural MRI studies discovered that brain structural changes associated with PPPD were primarily decreased cortical folding and GMV in many important brain regions. These changes were correlated significantly with disease duration and disease severity, suggesting that these brain structural changes may be important in the pathogenesis of PPPD. Some brain regions that show structural changes are parts of the parieto‐insular vestibular cortex (PIVC), including the STG, posterior insula, and ACC, among others. PIVC has been defined as the core of the multimodal vestibular cortex and is involved in processing and combining multi‐sensory inputs from the somatosensory, visual, and vestibular systems. Additionally, it plays a crucial role in processing data related to location, body posture, self‐motion, and external object movements (Eickhoff et al. 2006). Thus, structural changes in the PIVC region may indicate the important role of multi‐sensory integration dysfunction in the pathogenesis of PPPD.

However, because both studies were cross‐sectional, it is unclear whether the alterations in cortical folding and GMV are primary or secondary occurrences of PPPD. They may happen before PPPD as risk factors for the beginning of PPPD, or they could be the consequence of structural changes in the brain following the development of PPPD. Some researchers believe that the changes in brain function and structure in PPPD result from perceptual dysfunction rather than the cause of perceptual dysfunction. This perspective is still controversial and requires further confirmation research (Castro et al. 2022). In the future, we could enroll patients in the early stage of acute vertigo or dizziness. By comparing the structural MRI of the brain before and after the development of PPPD and comparing the structural MRI of the brain in patients who develop PPPD with those who do not, an answer to the above controversy may be found.

#### 2.5.2 Changes in Activity

In patients with CSD, fMRI was measured during sound‐evoked vestibular stimuli (Indovina et al. 2015). Compared to controls, CSD patients showed decreased activation to sound‐evoked vestibular stimulation in PIVC, including the anterior insula, posterior insula, hippocampus, IFg, and ACC. Reduced activation in these brain regions may be linked to persistent vestibular symptoms in CSD patients. In a later study by the same team, they studied fMRI response to examine changes in activity within the visual and vestibular cortices during self‐motion simulation in PPPD, which was achieved through visual virtual‐reality rollercoaster stimulation (Riccelli et al. 2017). They found that the anterior bank of the central insular sulcus showed increased activity in healthy controls during vertical motion compared to horizontal motion. However, this pattern was not observed in PPPD patients. For the same comparison, dizziness handicap positively correlated with increased activity in the visual cortex bilaterally in patients with PPPD. This could be the neural correlate of visual dependence. The above two studies indicate that the vestibular cortex of PPPD patients has a general down‐regulation of the activity or low responsivity to motion stimulation across vestibular and visual modalities. The alteration in cortical activity may be associated with the dizziness symptoms. However, these two studies did not distinguish PPPD patients as vestibular disease‐induced and non‐vestibular disease‐induced. There may be differences in the responsivity of the vestibular cortex to the above stimuli in PPPD patients with different causes. Therefore, further studies are required to confirm whether this change in cortical activity is universal in all types of PPPD. In a subsequent study, the same research group conducted a secondary analysis of the study above to further investigate the impact of personality traits on brain activity in PPPD patients (Passamonti et al. 2018). Neuroticism was found to increase the activity of neural networks that mediate attention to visual motion signals. The persistent symptoms following anxious status and visual exacerbation experienced by PPPD patients may be explained by these findings. The other fMRI study examined the brain activity of PPPD patients using emotionally charged visual stimuli (von Söhsten Lins et al. 2021). The results further confirmed the importance of emotional factors and visual stimuli in the pathogenesis of PPPD.

Additionally, Li et al. (2020b) discovered that PPPD patients had considerably lower amplitude of low‐frequency fluctuations in the right precuneus and cuneus compared to healthy controls. This suggests a reduction in spontaneous activity in this area. However, a recent study came to the opposite result (Liu et al. 2024). It reported increased fractional amplitude of low‐frequency fluctuation in the right precuneus in PPPD, which was associated with the degree of dizziness. The impaired function of the precuneus and cuneus may result in dysfunctional integration of vestibular and visual information, as the precuneus integrates vestibular and visual information (Cavanna and Trimble 2006) and the cuneus processes visuospatial information. The different results of the above two studies may be related to differences in the sample size, the duration of the disease, the severity of dizziness, and other factors. Moreover, Jiang et al. (2022) used magnetoencephalography to investigate activity changes in the brain in patients with PPPD. There was a significant increase in neuromagnetic activity in the temporal–parietal junction (TPJ) at 1–4 Hz and 4–8 Hz, and a significant increase was measured in the frontal cortex at 1–4 Hz. The study found a positive correlation between localized source strength in the TPJ at 1–4 Hz and the DHI score. The TPJ has bidirectional connections with the temporal and frontal lobes. It receives sensory inputs from visual, subcortical, auditory (Decety and Lamm 2007), and vestibular areas (Takeuchi et al. 2018). It is a crucial component of the PIVC and is responsible for integrating and processing multi‐sensory information (Eickhoff et al. 2006; Wurthmann et al. 2017).

These studies have shown that spontaneous and stimulus‐evoked activity in the cortex of PPPD patients is significantly different from that of healthy individuals and that this difference is correlated with dizziness symptoms. This provides a neurobiological basis for our understanding of the pathogenesis of PPPD. However, some of the research results are still controversial, and further clinical studies with larger sample sizes and fewer confounding factors are needed for further exploration.

#### 2.5.3 Changes in FC

FC alterations in different regions of the multimodal vestibular network were reported in patients with PPPD and its precursors. In the study of Indovina et al. (2015) using sound‐evoked vestibular stimulation, FC analysis revealed reduced connectivity between several brain regions in CSD patients, including the anterior insula and middle occipital gyrus, anterior insula and STG, hippocampus and STG, and ACC and STG. This indicates a change in the coordination of sensory and higher cortex regions. FC alterations between the middle occipital gyrus and the anterior insula may be linked to visual dependence in patients with CSD. Passamonti et al. (2018) reported that PPPD patients who scored high on neuroticism showed an increase in FC between the IFg and associative visual regions when exposed to vertical versus horizontal self‐motion simulation. This reflects that top‐down attention to vertical visual context may increase in neurotic patients. Similarly, a recent study also confirmed the effect of visual stimulation on FC in PPPD. After visual stimuli, there was an increased FC between the spatial cognitive and visual areas in PPPD patients (Yagi et al. 2023). This could contribute to the prolonged symptoms following a visual exacerbation in PPPD. These studies have shown that the FC changes between visual areas and other areas in PPPD patients after visual stimulation are opposite to those after vestibular stimulation. This indicates that the interaction of visual‐vestibular is reciprocal inhibitory. Additional research is needed to investigate the potential association between visual‐related symptoms and FC patterns in visual cortical regions in PPPD patients.

In the study by Li et al. (2020b), decreased FC among the cuneus, precuneus, and left precentral gyrus was reported. The weakened FC between these areas suggested the impaired ability to use vestibular and visual information to control posture and movement in patients with PPPD. The same team studied changes in intra‐ and inter‐network in PPPD (Li et al. 2020a). PPPD patients showed reduced intra‐network FC within the posterior default mode network in the right precuneus. Furthermore, seed‐based FC analysis revealed a decrease in intra‐network FC between the right precuneus and the bilateral precuneus and left premotor cortex and an increase in FC between bilateral corpus callosum and the right precuneus. In terms of inter‐network, there was an increase in FC between the occipital pole visual network and lateral visual and auditory networks, as well as the auditory and sensorimotor networks in PPPD patients. Moreover, there was a negative correlation between FC changes and DHI functional scores. These two studies showed that the precuneus of PPPD patients was functionally impaired and generated functional compensation through changes in intra‐ and inter‐network FC.

These important findings above indicate significant differences in FC between PPPD patients and healthy controls. However, the study by Lee et al. (2018) found that mental states could affect the FC among relevant brain regions in PPPD patients. In their study, the left hippocampal region, responsible for egocentric spatial navigation, showed a widespread reduction in FC in patients with PPPD compared to healthy controls. Increased FC between the subcallosal cortex and both the left middle frontal gyrus and the left superior lateral occipital cortex were shown in PPPD. However, the significance of the increased FC disappeared when controlling for psychiatric comorbidities. In the previous studies on FC in PPPD patients, the mental states of the control group rarely matched that of the experimental group, and the sample size of the studies was very small, which made the research results biased. Therefore, before the experiment begins, applying standardized psychological assessment tools to evaluate the mental states of the subjects and including PPPD patients and control groups with specific mental states will help eliminate the influence of mental states on FC and improve the accuracy of the research results.

#### 2.5.4 Changes in Perfusion

A study assessed the rCBF of PPPD patients using single photon emission computed tomography (Na et al. 2019). Patients with PPPD showed a significant rCBF decrease in the insula and frontal lobe compared to the control group, especially in the left posterior insula, right IFg, right precentral gyrus, left medial orbital gyrus, and bilateral superior frontal gyrus. In addition, PPPD patients showed a noteworthy increase in rCBF in the cerebellum bilaterally compared to the control group. This may be associated with increased demands on the cerebellum for postural control and visual attention. There was no correlation between changes in rCBF and the severity or duration of dizziness. However, this is the first study to evaluate cerebral perfusion in PPPD patients using single photon emission computed tomography, and there are no similar studies to verify its conclusion. The same team also studied the effect of transcranial direct current stimulation on rCBF in PPPD patients (Im et al. 2022). In their study, a noteworthy treatment‐by‐time effect on rCBF in the right superior temporal areas and left hippocampus was observed in the brain single photon emission computed tomography analysis. Therefore, transcranial direct current stimulation is a potential treatment for PPPD.

#### 2.5.5 Shifts in Multi‐Sensory Integration

The integration of vestibular, visual, and proprioceptive afferent information is necessary for normal posture and balance regulation. Any disruption in these systems can cause static or dynamic abnormalities. Several neuroimaging studies have demonstrated that patients with PPPD exhibit structural or functional changes in the brain regions responsible for multi‐sensory integration (Lee et al. 2018; Li et al. 2020a, 2020b; Na et al. 2019; Nigro et al. 2019; Riccelli et al. 2017; Wurthmann et al. 2017). As a result, the processing of spatial orientation information in PPPD patients relies on visual rather than vestibular and somatosensory inputs. In addition, the higher cortex is unable to regulate the processing of visual, vestibular, and somatosensory inputs, which induces the disorder of “sensory weighting” (Yamaguchi et al. 2022). Multi‐sensory integration dysfunction is considered one of the possible pathogenesis of PPPD and has been systematically investigated. Patients with PPPD performed significantly worse on sensory integration tests than healthy controls (McCaslin et al. 2022; Söhsten, Bittar, and Staab 2016). This suggests that they have difficulties with postural control in the face of multi‐sensory challenges. Moreover, PPPD patients showed greater medial–lateral sway in low‐demand conditions and greater anterior–posterior sway associated with visual dependence than controls (McCaslin et al. 2022). Anterior–posterior sway can distinguish PPPD from controls with high sensitivity and specificity under certain conditions of sensory integration tests. However, the results of studies on multi‐sensory integration dysfunction in people with PPPD lack information on more accurate quantitative sensory and perceptual tests. It is hoped that there will be more authoritative sensory and perceptual tests to quantify the influence of multi‐sensory integration dysfunction and help plan rehabilitation strategies.

In addition, when exposed to repeated pain stimuli, patients with PPPD showed lower pain habituation compared to controls (Holle et al. 2015). This suggests that damage to multi‐sensory afferent systems, such as pain afferent systems, may also exist in the pathogenesis of PPPD. More large clinical studies are needed to explore the parameter changes of PPPD related to multi‐sensory integration dysfunction. This may lead to the discovery of more meaningful diagnostic markers.

The results above offer a fresh perspective on the underlying pathophysiology of the disease. Perhaps PPPD is not a functional disorder without positive laboratory test results. It is just that we have not discovered these positive laboratory outcomes in the past. However, the correlation of structural and functional changes with the degree of dizziness is still controversial. It is meaningful to further clarify the causal relationship between these positive results and dizziness symptoms. Understanding the structural and functional changes in the brain associated with PPPD is a major challenge for a better definition of the disorder and the development of new therapeutic approaches. These changes may become imaging biomarkers of PPPD.

### 2.6 Other Potential Pathogenesis

#### 2.6.1 Impairment in Spatial Navigation Abilities

Determining and maintaining a trajectory among multiple points in an environment is called spatial navigation. It is a skill that develops from the effective management of an internal spatial map. Spatial navigation has been validated in both animal models and humans using the Morris water maze experimental paradigm. In addition, its defect has been shown to be a novel marker of cognitive impairment in Alzheimer disease (Wood 2016).

Breinbauer et al. proposed that impairment in spatial navigation abilities led to an improper perception of the environment and resulted in the symptoms of PPPD. They conducted three different virtual Morris water maze spatial navigational tasks to determine performance in PPPD patients. The PPPD group performed worse than the vestibular controls and healthy controls in the invisible target/navigationally demanding tasks, evidencing a remarkable impairment in spatial navigational abilities. Furthermore, PPPD patients also showed qualitatively different navigational behavior as they wandered in a disoriented, disorganized, and non‐strategic fashion without a clear focus point (Breinbauer et al. 2019).

Another of their studies found that spatial navigation and executive functions were uniquely impaired in PPPD patients. Factor analysis revealed that advanced visuospatial functions and spatial navigation were central to PPPD. They were strongly correlated with symptom severity (Breinbauer et al. 2023). Navigational performance allowed us to distinguish PPPD patients from non‐PPPD in challenging conditions. The findings above highlight the great potential of spatial navigation as a new diagnostic biomarker of PPPD. They also provide new perspectives on the pathophysiology of PPPD. The robustness of these findings needs to be assessed in larger and more diverse cohorts to allow for generalization to the wider PPPD population.

#### 2.6.2 Changes in the Vestibular Perception Thresholds

Vestibular thresholds are considered the most direct measure of vestibular perception (Kobel et al. 2021; Merfeld 2011). They reflect the ability of the brain to use peripheral vestibular input and central processing of afferent signals to generate percepts of self‐motion and awareness of head‐in‐space. Previous studies have demonstrated that vestibular perceptual thresholds are reduced in vestibular migraine, a condition that shares many common pathogeneses with PPPD (King et al. 2019; Lewis et al. 2011a, 2011b). Therefore, reduced vestibular perceptual thresholds are also the potential pathogenesis of PPPD. A cross‐sectional study tested motion perception thresholds and vestibular–ocular reflex thresholds to rotation by having subjects sit in a motorized rotary chair shielded from visual and auditory stimuli (Wurthmann et al. 2021). PPPD patients showed a significantly reduced vestibular perception threshold with increased motion sensitivity to the perception of rotary motion. The duration of the disease was found to be correlated with the extent of increased vestibular sensitivity. Decreased vestibular thresholds and increased vestibular excitability may lead to motion intolerance and dizziness induced by movement. However, because the study included only female participants, the effect of selection bias on the findings cannot be ruled out. In addition, not all states showed decreased vestibular perception thresholds. A case–control study found higher thresholds for roll tilt and superior–inferior *z*‐translations thresholds in PPPD patients, with the relative change in each threshold affected by the co‐occurrence of vestibular migraine (Kobel et al. 2024). Perceptual signals related to gravity may be impaired in patients with PPPD. Recently, Storm et al. (2024) assessed vestibular perception by galvanic vestibular stimulation and passive chair rotation in PPPD patients (Helmchen et al. 2024). Patients had a significantly lower vestibular perception threshold during galvanic vestibular stimulation, which appears to facilitate postural misperception and instability. However, vestibular perception thresholds were indistinguishable during passive vestibular chair rotation in the darkness.

The studies above illustrate that PPPD patients may show different changes in vestibular perception thresholds when facing motions in different directions. However, behavioral studies into vestibular perception are scarce currently. Furthermore, these studies did not rule out the influence of anxiety states, which could also reduce vestibular thresholds (Balaban and Thayer 2001). More well‐designed studies are needed to investigate the characteristics of vestibular thresholds in PPPD patients. Vestibular thresholds are specific, sensitive, and reliable methods to measure vestibular perception. Therefore, vestibular thresholds are expected to be powerful tools for diagnosing PPPD and evaluating rehabilitation efficacy.

#### 2.6.3 Central Sensitization: a Possible Exacerbating Factor

Central sensitization is a neurophysiological condition in which the central nervous system becomes over‐excitable (Woolf 2011). It has been reported that central sensitization syndrome is associated with several functional diseases, such as migraine, tension headache, fibromyalgia, rheumatoid arthritis, and irritable bowel syndrome. Central sensitization cannot be directly measured, and it is usually assessed using the Central Sensitization Inventory (Tanaka et al. 2017).

The relationship between central sensitization and PPPD has been investigated. The coexistence of central sensitization syndrome in PPPD may exacerbate the level of disability caused by dizziness symptoms and lead to a higher rate of complications of depression and anxiety (Hashimoto et al. 2022). In addition, the regression analysis revealed that central sensitization may be an exacerbating factor of PPPD. Therefore, psychosomatic medicine may be a significant component of the treatment of PPPD.

#### 2.6.4 Oxidative Stress

“Oxidative stress” is a concept developed in the field of redox biology and medicine about 40 years ago. The high level of energy consumption in the brain makes it more susceptible to oxidative stress than any other organ in the body. Therefore, oxidative stress plays a significant role in the pathogenesis of many nervous system diseases.

Fang et al. (2020) investigated the pathophysiologic mechanisms of oxidative stress in CSD patients using serum samples. Their research is the first to explore the characteristics of the redox system in individuals with CSD compared to a healthy population. They found that oxidative stress parameters, such as hydrogen peroxide and reactive substances, were significantly elevated in CSD. In contrast, activities of endogenous antioxidant components such as catalase and superoxide dismutase were considerably reduced in the CSD group. Additionally, the degree of subjective dizziness had a strong negative correlation with total glutathione and Trolox equivalent antioxidant capacity. These findings suggest that oxidative stress is closely related to the occurrence and severity of PPPD. Oxidative stress‐related parameters can be diagnostic biomarkers of PPPD. Moreover, this indicates that antioxidant therapy may be an effective treatment for PPPD. The pathophysiological mechanisms associated with oxidative stress need to be further investigated in patients who meet the diagnostic criteria of PPPD.

## 3 Conclusions and Future Directions

PPPD is a functional disease with high incidence, which comes to be a real burden for the work and life of the patient. However, due to the lack of pathognomonic findings on physical examination, laboratory testing, or diagnostic imaging for PPPD, it is difficult to create animal models and conduct molecular pathology studies and drug trials related to the pathogenesis of PPPD. As a result, progress in the diagnosis and treatment of PPPD has been relatively slow. Fortunately, with the rapid development of neuroimaging and molecular biology, our understanding of the pathogenesis of PPPD has gradually improved. In this review, we provide a comprehensive update on the emerging understanding of the pathogenesis of PPPD.

Currently, there are no definitive guidelines for treating PPPD. A clearer understanding of its pathogenesis can enhance treatment strategies. For instance, cognitive behavior therapy combined with anti‐anxiety medication can be beneficial for PPPD patients experiencing significant anxiety, as it not only reduces anxiety but also boosts medication efficacy (Yu et al. 2018). Cognitive behavior therapy is also effective in counteracting misperceptions of motion. Vestibular rehabilitation therapy has been widely used for PPPD patients. In the future, personalized vestibular rehabilitation programs tailored to different postural control strategies and vestibular perception thresholds may prove more effective than standard approaches. For PPPD patients with altered brain activity, neuromodulation therapies like transcranial direct current stimulation (Im et al. 2022) or repetitive transcranial magnetic stimulation (Staab 2023) show promise. However, due to a lack of sufficient clinical studies, their effectiveness is uncertain. Future research should focus on comparing the effects of different stimulation targets, frequencies, and treatment durations to identify the most suitable and effective treatment plans. Particularly, discovering the optimal multimodal treatment strategy will be a key area of future research.

## Author Contributions

**Chen Qin**: conceptualization, writing–original draft, writing–review and editing, investigation, validation, supervision, project administration. **Ruyi Zhang**: writing–review and editing, software. **Zhihui Yan**: project administration, supervision, conceptualization, writing–review and editing.

## Ethics Statement

The authors have nothing to report.

## Conflicts of Interest

The authors declare no conflicts of interest.

### Peer Review

The peer review history for this article is available at https://publons.com/publon/10.1002/brb3.70229

## Data Availability

Data sharing is not applicable to this article as no datasets were generated or analyzed during the current study.
